# Protective hybrid coating containing silver, copper and zinc cations effective against human immunodeficiency virus and other enveloped viruses

**DOI:** 10.1186/s12866-016-0675-x

**Published:** 2016-04-01

**Authors:** Jan Hodek, Veronika Zajícová, Irena Lovětinská-Šlamborová, Ivan Stibor, Jana Müllerová, Jan Weber

**Affiliations:** Institute of Organic Chemistry and Biochemistry AS CR, Flemingovo nam. 2, 166 10 Prague 6, Czech Republic; Department of Chemistry, Faculty of Science, Humanities and Education, Technical University of Liberec, Studentska 1402/2, 461 17 Liberec 1, Czech Republic; Institute of Health Studies, Technical University of Liberec, Studentska 1402/2, 461 17 Liberec 1, Czech Republic; Centre for Nanomaterials, Advanced Technology and Innovation, Bendlova 1407/7, 461 17 Liberec 1, Czech Republic

**Keywords:** Hybrid coating, Virucidal effect, HIV, Enveloped viruses

## Abstract

**Background:**

Healthcare-acquired infections by pathogenic microorganisms including viruses represent significant health concern worldwide. Next to direct transmission from person-to-person also indirect transmission from contaminated surfaces is well documented and important route of infections. Here, we tested antiviral properties of hybrid coating containing silver, copper and zinc cations that was previously shown to be effective against pathogenic bacteria including methicillin-resistant *Staphylococcus aureus*. Hybrid coatings containing silver, copper and zinc cations were prepared through radical polymerization via sol-gel method and applied on glass slides or into the wells of polymethylmethacrylate plates. A 10 μl droplet of several viruses such as human immunodeficiency virus type 1 (HIV-1), influenza, dengue virus, herpes simplex virus, and coxsackievirus was added to coated and uncoated slides or plates, incubated usually from 5 to 240 min and followed by titer determination of recovered virus.

**Results:**

Scanning electron microscopy analysis showed better adhesion of coatings on glass surfaces, which resulted in 99.5–100 % HIV-1 titer reduction (3.1 ± 0.8 log_10_TCID_50_, *n* = 3) already after 20 min of exposure to coatings, than on coated polymethylmethacrylate plates with 75–100 % (1.7 ± 1.1 log_10_TCID_50_, *n* = 3) and 98–100 % (2.3 ± 0.5 log_10_TCID_50_, *n* = 3) HIV-1 titer reduction after 20 and 120 min of exposure, respectively. Slower virucidal kinetics was observed with other enveloped viruses, where 240 min exposure to coated slides lead to 97 % (dengue), 100 % (herpes simplex) and 77 % (influenza) reduction in virus titers. Interestingly, only marginal reduction in viral titer after 240 min of exposure was noticed for non-enveloped coxsackie B3 virus.

**Conclusions:**

Our hybrid coatings showed virucidal activity against HIV and other enveloped viruses thus providing further findings towards development of broad-spectrum antimicrobial coating suitable for surfaces in healthcare settings.

## Background

Healthcare–associated infections (HAIs) by pathogenic bacteria, viruses and other microorganisms constitute significant cause of morbidity and mortality worldwide. Although exact statistical data are lacking, 1.7 million HAIs were estimated to occur in 2002 in U.S. hospitals alone [1]. More recent survey indicates that everyday approximately 1 of every 25 patients in U.S. acute care hospitals has at least one HAI [2]. Next to direct transmission from person-to-person indirect transmission from contaminated surfaces is also well documented and important route of infections. Self-decontaminating surfaces would represent additional safety measure towards preventing transmission in healthcare settings. In recent years a growing interest is seen in the use of organic-inorganic hybrid materials in medicine, for example antibacterial protective coatings applied on number of devices used in healthcare [3–7], micro and/or nano needles for transdermal drug delivery [8], antibacterial protective coatings on the surface of artificial joints [9], or dental implants [10].

Hybrid materials are very conveniently prepared by sol-gel method, which can be briefly described as a special process of glass and ceramics manufacture at room or slightly elevated temperature. This technique became an independent and a very comprehensive discipline, documented by numerous books [11–15] and reviews [16, 17]. With the introduction of organically-modified silicates (ORMOSILs) the sol-gel products started to be an interesting precursor for number of hybrid materials applicable in medicine. Specifically 3-(trimethoxysilyl)propyl methacrylate (TMSPM) either in the pure form or in addition to tetrafunctional silica precursors such as tetraethylorthosilicate (TEOS) or tetramethylorthosilicate (TMOS) allows large variations in optical, mechanical and chemical properties and can be tailored for specific uses [18, 19]. In addition, the sol-gel process allows the encapsulation of different nanoparticles with known antimicrobial activity e.g. metallic nanoparticles such as Ag, Cu, Zn, Au etc. [4, 20].

From metal nanomaterials with biocidal properties, silver and copper received the most attention. Next to well documented bactericidal activities both silver and copper showed potent virucidal properties [21]. Several studies have confirmed anti-HIV activity of silver nanoparticles [22–24] inhibiting most likely entry step by binding directly with gp120 [25]. Furthermore, silver nanoparticles were shown to inhibit herpes simplex virus type 1 [26] and 2 [27], vaccinia virus [28], respiratory syncytial virus [29], influenza A [30], tacaribe virus [31] and hepatitis B virus [32].

Virucidal activity of copper in the form of copper oxide was evaluated in variety of materials such as fibers, latex, filter matrices and other polymeric materials [33, 34]. Copper oxide-containing filters effectively neutralized HIV-1 in medium and breastmilk and reduced cell-associated HIV in dose-dependent manner [35, 36]. In addition, these filters reduced infectious viral titers of several DNA and RNA viruses, among others yellow fever virus, influenza A virus, measles virus, respiratory syncytial virus, adenovirus type 1 and cytomegalovirus [37]. Recently, it was shown that cuprous compounds deactivate more efficiently bacteriophages and bacteria than cupric compounds [38].

Photoactivation of titanium dioxide by UV generates reactive oxygen molecules on the surface of TiO_2_ and has been shown to effectively inactivate influenza A virus [39], HIV-1 [40], and murine norovirus [40, 41]. Halogen and interhalogen TiO_2_ nanoparticles, except for chlorinated adduct, completely inactivated bacteriophages MS-2, ψ-X174 and PRD-1, showing that oxidizing potential can be generated without UV photoactivation [42]. Similarly, metal oxide nanoparticles, CeO_2_ and Al_2_O_3_, and their halogen adducts exhibited excellent virucidal activities against bacteriophages.

In addition to virucidal metal nanoparticles, polymeric coatings with antiviral activities were reported. Coatings based on hydrophobic polycations N,N-dodecyl methyl-polyethylenimine on a glass slide lowered influenza infectious titer by at least four fold [43]. Similarly, up to five fold reduction of influenza infectious virus titer was achieved with hyperbranched polymers with quaternary ammonium on glass surfaces, while no significant reduction of infectious poliovirus was detected [44].

Recently, we have reported on new type of hybrid coating containing silver and copper cations with strong antibacterial effect against variety of commonly occurring bacteria in hospital including *Staphylococcus aureus* and its methicillin-resistant variant [6]. Here, we have tested antiviral properties of this type of hybrid coating.

## Methods

### Chemicals

Tetraethylorthosilicate (TEOS, Sigma-Aldrich, 98 wt.%), 3-(trimethoxysilyl)propyl methacrylate (TMSPM, Sigma–Aldrich, 98 wt.%), methylmethacrylate (MMA, Sigma-Aldrich, 99 wt.%), dibenzoylperoxide (BPO, Luperox® A75, Sigma-Aldrich, 75 wt.% and 25 wt.% of water for stabilisation), titanium isopropoxide (IPTI, Sigma-Aldrich >97 wt.%), silver nitrate (AgNO_3_, Sigma-Aldrich, 99.8 wt.%), copper nitrate trihydrate (Cu(NO_3_)_2_.3H_2_O, Sigma-Aldrich, 98–103 wt.%), zinc nitrate hexahydrate (Zn(NO_3_)_2_.6H_2_O, Sigma-Aldrich, 98 wt.%), propan-2-ol (Penta CZ, p.a. 99.98 wt.%), nitric acid (HNO_3_, Lach:ner CZ, p.a. 65 wt.%), distilled water, photoinitiator Irgacure 819 (Ciba CZ, 45 wt.%). Substrates used for preparation of hybrid coatings; glass covering, size 18 × 18 mm with thickness 0.13 – 0.17 mm, (INTRACO MICRO, CZ); poly(methyl methacrylate) – Cell Culture Plates (PMMA), size 75 × 125 × 15 mm, (Corning Incorporated NY, Costar, USA).

### Sol synthesis

Silver nitrate (0.12 g, 0.71 mmol) was dissolved in 26 ml of propan-2-ol by stirring at room temperature for 60 min. Subsequently, TEOS (1.9 ml, 8.51 mmol), TMSPM (1.0 ml, 4.21 mmol), MMA (0.9 ml, 8.41 mmol) and BPO (0.1 g, 0.41 mmol) were added via septum into the flask and the reaction mixture was stirred under the atmosphere of nitrogen at room temperature until all of the BPO dissolved. After that, 0.2 ml of a 2 M solution of nitric acid, distilled water (0.4 ml, 22 mmol), copper nitrate trihydrate (0.1 g, 0.40 mmol) and zinc nitrate hexahydrate (0.11 g, 0.37 mmol) were dissolved in 26 ml of propan-2-ol in a separate flask and this solution was added to the sol and stirred intensively at room temperature.

Finally, IPTI (0.6 ml, 2.22 mmol) was added and the reaction mixture was stirred for another 15 min at room temperature. The resulting sol was heated to reflux in an oil bath while being stirred for 35 min, then cooled down to room temperature and stored in a polyethylene bottle in the dark at 20 °C. A sol prepared in this manner was used within three weeks.

### Preparation of substrates for application of coatings

Glass samples were mechanically cleaned with a commercial detergent (Jar), then rinsed with distilled water, sonicated in the same medium in an ultrasound bath for 5 min and rinsed with distilled water. Then, they were immersed in nitric acid diluted 1:1 with distilled water for three min, repeatedly rinsed with distilled water and finally with propan-2-ol. The cleaned substrates were stored in propan-2-ol. PMMA samples were washed with a commercial detergent (Jar), then rinsed with distilled water several times and immediately immersed in propan-2-ol in an ultrasound bath for 5 min. Finally, they were rinsed with propan-2-ol and stored in it.

### Coating procedures

#### Dip-coating

The glass substrates were dipped into sol, then were withdrawn at a speed of 4 cm.min^-1^.

#### Flow-coating

The sol diluted with 1 % solution of the photoinitiator Irgacure 819 (BASF, Switzerland) in molar ratio 1:1 (300 μl) was dropped into wells in PMMA plates by calibrated pipette (V = 300 μl). The solution was left in the wells for 30 s, and subsequently was removed by the same pipette from each well.

#### Curing

The coatings on glass were left for 60 min at room temperature, then were cured for 3 h at 150 °C. The coatings on PMMA plates were left for 60 min at room temperature, then were cured for 1 h at UV-A (315–400 nm, Philips Actinic BL 15 W, made in Holland). The distance of fluorescent lamp from samples was 30 cm.

### Measuring properties of coatings

#### FT- IR spectroscopy

The measurements of reflective IR spectra were performed on an FT-IR spectrometer Nicolet™ iSTM10 (Thermo Scientific™) at room temperature (25 °C). The spectrometer was used with the extension method ATR crystal Ge. For liquid samples, the measurements were performed after evaporation of the solvent. The solid samples were measured on a thin layer of aluminum foil.

### Surface morphology – scanning electron microscopy

The quality and morphology of the hybrid coatings was measured through Scanning Electron microscope Carl Zeiss ULTRA Plus with micro-analytic fragment EDS system Silicon Drift Detector 20 mm^2^ (SDD) - X-you max (OXFORD Instruments). The transparent samples (magnified 1120x and 18280x) were gold-dusted 3 nm through QR150R (Quorum Technologies) with ion evaporation system model 1060 SEM Mill (Fischione) and observed (through the In-Lens detector) in the form of secondary electrons SE1.

### Cells and viruses

All cells were mycoplasma negative (routinely tested at Generi Biotech, Czech Republic) and cultured in tissue-culture treated polystyrene plates in 5 % CO2 and at 37 °C. TZM-bl cells (Dr. John C. Kappes, Dr. Xiaoyun Wu and Tranzyme Inc.) and HeLa cells (Dr. Richard Axel) were obtained through the NIH AIDS Reagent Program, Division of AIDS, NIAID, NIH. Madin-Darby canine kidney cells (MDCK) and HB-46 cells were obtained from the American Type Culture Collection (ATCC, Manassas, VA), Vero cells from the European Collection of Cell Cultures and HEK293T cells from Stanford University (Stanford, CA). All cells were maintained in DMEM with L-glutamine, 10 % fetal bovine serum (FBS), 100 U of penicillin/ml and 100 μg of streptomycin/ml (all Sigma-Aldrich, St. Louis, MO). Virus HIV-1_NL4-3_ was prepared by transfection of plasmid pNL4-3, obtained through the NIH AIDS Reagent Program, Division of AIDS, NIAID, NIH from Dr. Malcolm Martin, into HEK293T cells and after 48 h supernatant was harvested, filtered through 0.45 μm steri-flip, aliquoted and stored at -80 °C. Human coxsackie B3 virus (strain Nancy) and human herpesvirus 1 (strain HF) were obtained from ATCC, influenza virus H1N1 A/Mexico/4108/2009 from Diagnostic Hybrids (Athens, OH) and dengue virus type 2 from Dr. Jochen Bodem, University of Wurzburg (Wurzburg, Germany).

### Experimental setup for determination of virucidal activity

#### Cover glass experiments

Coated or uncoated (in control experiment) 18 × 18 mm cover glasses were placed into 6-well plate and 10 μl droplet of 1.66 × 10^5^ 50 % tissue cell culture infectious doses (TCID_50_)/ml of HIV-1 or 3.63 × 10^5^ TCID_50_/ml of influenza A/H1N1 virus or 3.98 × 10^6^ TCID_50_/ml of dengue virus type 2 virus or 1.00 × 10^6^ TCID_50_/ml of herpes simplex type 1 virus (HSV-1), or 3.16 24 × 10^4^ TCID_50_/ml of coxsackie B3 virus was applied in the center of the glass. The droplet was immediately covered by uncoated cover glass to spread the virus on the whole area. After incubation at specified time usually 5, 10, 20, 30, 60, 120, and 240 min, 490 μl of 1X phosphate buffered saline (PBS) was added to the coverslips, the top coverslip was lifted and the virus-exposed sides of both coverslips were washed by pipetting 3 times. We collected the whole washing and used for titer determination. Experiments were performed with three cover glasses for each time point. All virus titer determinations of recovered virus were performed with 11 twofold dilutions in triplicate according to the type of virus as mentioned below and virus titer reduction was expressed in percentage or plotted as TCID_50_ [IU/ml] versus time.

### Experiments with 96-well plates

Virus droplet (10 μl) of 1.66 × 10^5^ TCID_50_/ml of HIV-1 was placed in the center of wells (coated with photopolymerization and uncoated) in 96-well plate and covered with lid. At 2, 5, 10, 20, 30, 60, and 120 min 90 μl of 1X PBS was added to the well, mixed three times by pipetting up and down and removed from the well. All experiments in 96-well plates were performed in triplicate. Recovered virus was immediately titrated according the protocols mentioned below and virus titer reduction was expressed in percentage.

### Titer determination

The determination of all virus titers were performed in triplicate from twofold serial virus dilutions and calculated according the method by Reed and Muench [45]. Methods for the discrimination of infected and uninfected wells were virus specific and are described below.

### HIV titer determination

TZM-bl indicator cell line was used to quantitate HIV titers [46]. Twofold serial dilutions of HIV were added in triplicate to 30,000 TZM-bl cells plated 24 h before in DMEM with L-glutamine, 10 % fetal bovine serum, 100 U of penicillin/ml and 100 μg of streptomycin/ml. After 48 h incubation at 37 °C in 5 % CO_2_, the supernatant was removed, cells washed with 1X PBS, fixed with 1 % glutaraldehyde and incubated with X-galactosidase staining solution. After 2 h of incubation infected wells that developed blue loci were counted as positive. Alternatively, after 48 h of incubation the firefly luciferase luminescent assay was performed and luminescence was measured in Victor X3 plate reader (Perkin Elmer, Waltham, MA). Wells with relative light units above background (mean of luminescence of negative wells plus two standard deviations) were considered positive.

### Dengue virus titer determination

Immunofluorescence staining was used to visualize infected cells [47]. Briefly, twofold serial dilutions of dengue virus type 2 were added in triplicate to 20,000 Vero cells plated day before in DMEM with 2 % fetal bovine serum, 100 U of penicillin/ml and 100 μg of streptomycin/ml. After 72 h of incubation at 37 °C in 5 % CO_2_, cells were fixed with 4 % paraformaldehyde and permeabilized with 0.2 % Triton X-100. Then, cells were washed with 1X PBS and incubated overnight at 4 °C with dengue virus type 2 serotype-specific mouse monoclonal antibody, which was harvested from HB-46 cells. Wells were washed three times with 1X PBS, incubated 90 min with Cy3-labeled donkey anti mouse IgG (Jackson Immunoresearch Europe) and documented using fluorescence microscope Olympus IX-81 with camera (Hamburg, Germany). ImageJ software (NIH) was used for image analysis and evaluation of positive and negative wells.

### Influenza virus titer determination

Immuno-stained plaque assay using low-viscosity overlay medium developed by Matrosovich et al. [48] was adapted for influenza virus determination. Day before 25,000 MDCK cells were plated in DMEM medium with 10 % FBS. Following day cells were washed twice with 1X PBS, replenished with DMEM medium without FBS and twofold serial dilutions of influenza virus were added to the cells. After 1 h of incubation the cells and virus mixture was overlaid with 1:1 mixture of 2.4 % Avicel RC/CL (FMC Biopolymer, Philadelphia, PA) and DMEM without FBS, with 7.5 % bovine serum albumine, 0.5 μg/ml TPCK-trypsin (both from Sigma-Aldrich), 100 U of penicillin/ml and 100 μg of streptomycin/ml. After one day of incubation in 37 °C in 5 % CO_2_, the avicel solution was removed from the wells, the cells were washed with 1X PBS, fixed with 4 % paraformaldehyde, permeabilized with 20 mM glycine/0.5 % Triton X-100 for 20 min and incubated for 1 h with 1:2500 dilution of monoclonal influenza A antibody against nucleocapsid (Merck Millipore, Darmstadt, Germany) in 10 % normal horse serum and 0.05 % Tween 80 (both Sigma-Aldrich). Mouse secondary antibody (1:5000 dilution) in 10 % normal horse serum and 0.05 % Tween 80 was applied for 1 h and followed by 30 min of incubation in TrueBlue (KPL, Gaithersburg, MA) until blue plaques developed in infected wells.

### Herpes simplex and coxsackie virus titer determination

Twofold serial dilutions of herpes simplex and coxsackie B3 virus were added to 20,000 Vero cells and 30,000 HeLa cells, respectively. Both cell lines were plated day before in DMEM with 10 % FBS and following day the medium was replaced with serum-reduced DMEM with 2 % FBS. After 1 h of incubation, the cells and virus mixture was overlaid with 1:1 mixture of 2.4 % Avicel RC/CL and DMEM with 2 % FBS. After three days of incubation in 37 °C in 5 % CO_2_ the same procedure was followed as with influenza titer determination with following changes in the virus specific antibody dilutions. HSV-1 infected cells were incubated with 1:800 dilution of anti-HSV type I and II monoclonal antibody and coxsackievirus-infected cells were incubated with 1:1000 dilution of anti-coxsackievirus B3 antibody (both Merck Millipore, Darmstadt, Germany).

### Measurement of cytotoxicity of antiviral coatings

Six uncoated and twelve coated glass covers (half of the covers were prepared by photopolymerization and half were polymerized by heat) were incubated with 500 μl of 1X PBS for 15 sec and for 4 h. After incubation, 50 μl of liquid were transferred in triplicate on HeLa and Vero cells, incubated for 72 h and cytotoxic effect was analyzed by XTT colorimetric assay. Briefly, 50 μl of 50:1 mixture of XTT labeling reagent (1 mg/ml) and PMS electron-coupling reagent (0.383 mg/ml) was added to the wells and incubated for 4 h in 37 °C in 5 % CO2. Formation of orange formazan dye was measured in Victor X3 plate reader and wells with absorbance lower than cutoff that was calculated as mean of absorbance of wells without virus plus two standard deviations were considered infected.

### Statistical analysis

Data were analyzed using a one-way analysis of variance test and followed by the Bonferroni’s multiple comparison test. All differences with a *p*-value less than 0.05 were considered statistically significant. Statistical analyses were performed using GraphPad Prism v.6.05 (GraphPad Software, La Jolla, CA).

## Results

### FT-IR spectroscopy

**Fig. 1:**
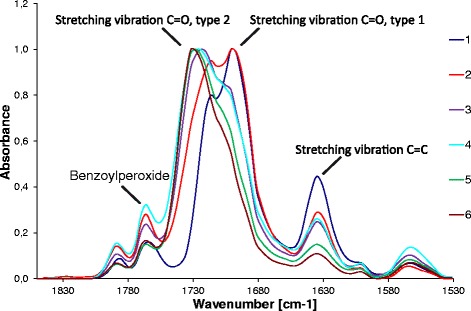
FT-IR; polymerization of the sol. Sampling in different time intervals (1- 5 min of sol reflux; 2 – 10 min of sol reflux; 3 – 15 min of sol reflux; 4 – 20 min of sol reflux; 5 – 25 min of sol reflux; 6 – 35 min of sol reflux)

Figure 1 shows the FT-IR spectra of the organic-inorganic sol obtained from the combination of TEOS, TMSPM, MMA, and IPTI in the presence of BPO. The characteristic stretching vibration peaks of -C = O (type 1 and type 2) occurred at 1660 – 1740 cm^-1^ for type 1, and 1655 – 1750 cm^-1^ for type 2. The infrared absorption of bimodal frequency distribution occurred due to the conjugation effect between -C = O and nearby -C = C- and hydrogen bonds. The characteristic stretching vibration peak of -C = C- occurred at 1633 cm^−1^ and the characteristic stretching vibration peak of BPO initiator occured at 1787 - 1767 cm^-1^. In Fig. 1 the progress of free radical polymerization of acrylates is visible (see also Table 1). The conjugated -C = C-C = O group is gradually transformed into -C-C-C = O and this change is acompanied by weakening of -C = C- band. At the same time the -C = O of BPO is weakening as well as this compound gradually decomposes. It confirms that TMSPM and MMA molecules were bound together and most likely also grafted on the SiO_2_ in the process of TEOS and TMSPM co-hydrolysis.

**Table 1:** FT-IR; a polymerization of the sol used for the preparation of hybrid coatings

Sample	Time	Stretching vibrations of C = O and C = C groups
1	5 min reflux	1787 – 1767 cm^-1^ stretching vibration -C = O bonds (BPO) 1660 – 1740 cm^-1^ stretching vibration -C = O bonds, type 1 (MMA) 1655 – 1750 cm^-1^ stretching vibration -C = O bonds, type 2 (propionate) 1633 cm^-1^ stretching vibration -C = C- bonds
2	10 min reflux	
3	15 min reflux	
4	20 min reflux	
5	25 min reflux	
6	35 min reflux	

### Surface morphology obtained through SEM

**Fig. 2:**
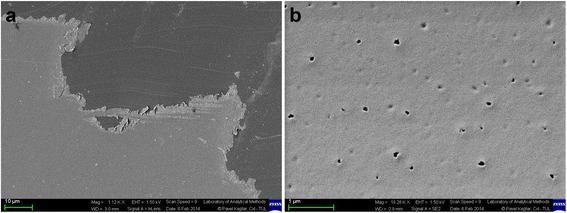
SEM images of PMMA substrates with hybrid coatings; **a** the edge of the hybrid coating; **b** the hybrid coating contains a large amount of pores in size of a few nm

**Fig. 3:**
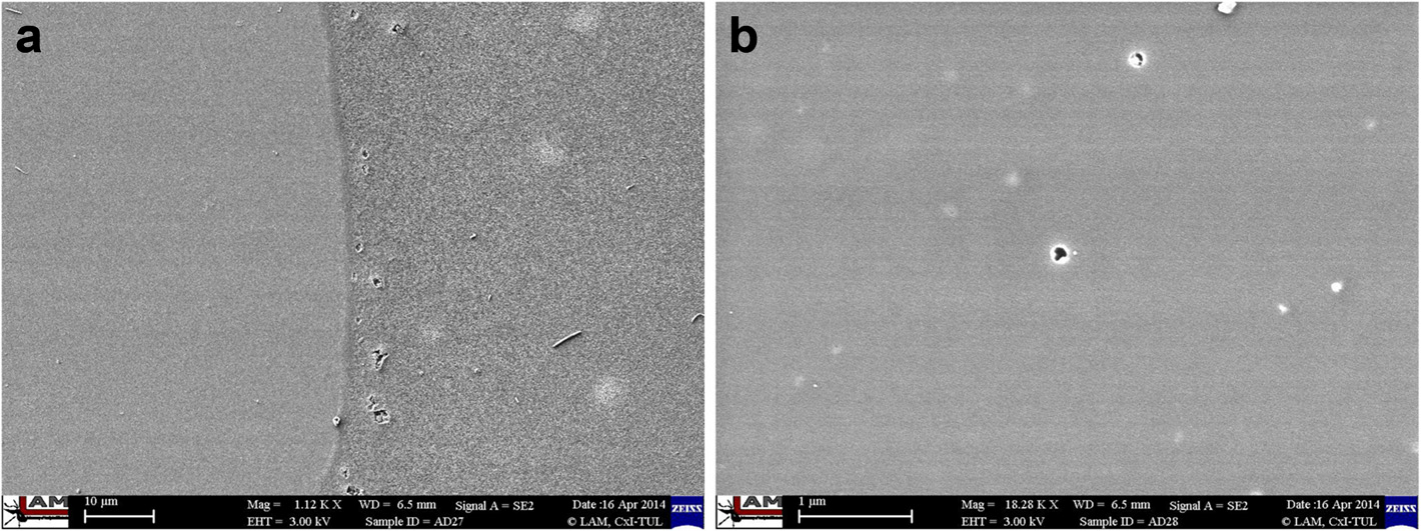
SEM images of glass substrates with hybrid coatings; **a** the hybrid coating on the right side of the sample; **b** the hybrid coating containing two pores in size of a few nm

The surfaces of hybrid coatings were analyzed using SEM on two types of substrates; PMMA plates (Fig. 2a, b) and glass (Fig. 3a, b). It confirmed that the hybrid coatings almost precisely copied the surface of the substrates (Fig. 2 and 3).

We observed a number of clearly visible pores in size of few nm on the coated PMMA sample (Fig. 2b), in contrary to that on the coated glass sample, where the number of pores was lower. This can be attributed to the different type of polymerization used. The photopolymerization with photoinitiator Irgacure 819 was used for coating PMMA samples via UV-A (range 315–400 nm). It was dictated by temperature sensitivity of PMMA that is not stable at temperature higher than 60 °C. The glass samples, however, were polymerized by heating for 3 h at 150 °C. The hybrid coating on the glass substrate (Fig. 3a, b) showed excellent adhesion to the surface. The adhesion of the hybrid coating to PMMA substrate was much weaker which might be improved by plasma treatment of PMMA surface prior coating.

### Cytotoxicity of hybrid coatings to Vero and HeLa cells

**Fig. 4:**
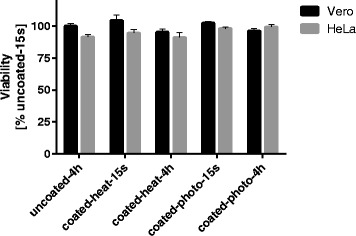
Effect of hybrid coating washings on Vero and HeLa cells proliferation activity. Vero (black bars) and HeLa (grey bars) cell lines were exposed to washing of coatings on glass coverslip prepared by photo- and heat polymerization after 15 sec and 4 h of incubation in 1X PBS. Cell viability after 15 sec of incubation with 1X PBS of uncoated glass coverslip was set as 100 % for each cell line. Error bars indicate standard deviations of results from three glass coverslips

The cytotoxicity of washings from hybrid coatings with silver, copper and zinc prepared by heat and photopolymerization were evaluated in Vero and HeLa cell lines at two time points (Fig. 4) Immediate cytotoxic effect was assessed after 15 sec exposure to coated glass and resulted in no loss of viability of Vero cells and only small (2–5 %) loss of viability of HeLa cells. Even exposure for four hours did not release any significant toxic material from coated glass coverslips, which is demonstrated by the viability values above 90 % for both cell lines and both polymerization methods. The lowest viability was observed with hybrid coatings prepared by heat polymerization with 95 % viability for Vero cells and 91 % viability for HeLa cells when compared to the viability of washings from uncoated glass slides after 15 sec exposure.

### Anti-HIV-1 activity of hybrid coatings on glass slides

As a first step in evaluating the virucidal activity of hybrid coatings we coated six glass coverslips by heat polymerization, six by photopolymerization and six were left uncoated. We applied 10 μl of HIV-1 on the coverslip and incubated three coverslips for 5 min and three coverslips for 20 min. The Table 2 displays recovered HIV-1 titer after washing with 0.49 ml of 1X PBS for each coverslip. We found no statistically significant difference in HIV-1 viability between 5 and 20 min exposure of HIV-1 to uncoated glass coverslips with HIV-1 titers of 3981 TCID_50_/ml and 2987 ± 1721 TCID_50_/ml, respectively. Moreover, recovered virus titer approximately matched initial input of HIV-1 (3320 TCID_50_/ml) after similar dilution in 0.49 ml of PBS without exposure to the uncoated glass coverslip. Decrease in HIV-1 titer was already noticeable after 5 min exposure to hybrid coatings. The decrease was smaller in the case of glass coverslips with hybrid coatings prepared by heat polymerization with 79 % HIV-1 titer reduction on average, than in the case of glass coverslips with hybrid coatings prepared by photopolymerization where the reduction was 98 %. Similarly, 20 min exposure of HIV-1 on glass coverslips with hybrid coatings prepared by photopolymerization exhibited better virucidal activity that resulted in 100 % inactivation of HIV-1 on two out of three coverslips than coverslips coated by heat polymerization where 10 % of residual HIV-1 activity remained.

**Table 2:** Anti-HIV-1 activity of hybrid coating on glass coverslips

	Exposure time [min]	HIV-1 titer [TCID_50_/ml] after exposure to uncoated and coated coverslip and after washing with 0.49 ml of PBS			HIV-1 titer reduction^*a*^	*p*-value^*b*^
		Coverslip #1	Coverslip #2	Coverslip #3		
Uncoated coverslip	5	3981	3981	3981	-	-
	20	3981	1000	3981	-	-
Coated coverslip by heat polymerization	5	251	1259	1000	79 %	<0.001
	20	316	251	316	90 %	<0.05
Coated coverslip by photopolymerization	5	63	63	63	98 %	<0.001
	20	0	20	0	99.8 %	<0.05

^*a*^HIV-1 titer reduction was calculated from average HIV-1 titers from uncoated coverslips set as 100 % minus the quotient between residual average HIV-1 titers from coated coverslips and average titers from uncoated coverslips expressed in percentage

^*b*^One way analysis of variance test between HIV-1 titers obtained from uncoated coverslips versus coated coverslips at given exposure time

### HIV-1 inactivation on coated PMMA surface

**Fig. 5:**
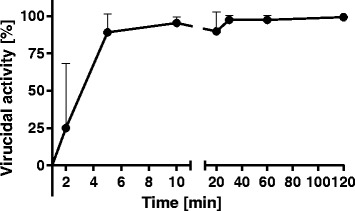
Time course of HIV-1 inactivation by hybrid coating on PMMA wells. Virucidal activity is expressed as HIV-1 titer reduction in percentage. Error bars indicate standard deviations of three independent experiments performed in three replicate wells for each time point

Next we examined the anti-HIV-1 activity of hybrid coating when applied on poly(methyl methacrylate) surface. Figure 5 summarizes results from three separate experiments each performed in triplicate. We can see quick virucidal effect in first 5 min followed by slower additional virucidal effect during the span of 2 h. Interestingly, complete virucidal efficiency was achieved in only one out of three experiments. Nevertheless, at least 98 % reduction of HIV-1 titer was obtained after 2 h of exposure to coated PMMA wells.

### Effect of hybrid coatings against other viruses

**Fig. 6:**
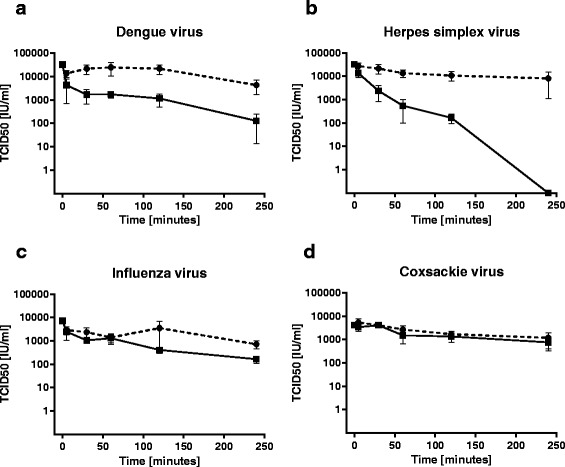
Time course of virucidal activity of hybrid coatings on glass coverslips against **a** dengue virus, **b** herpes simplex virus, **c** influenza virus, and **d** coxsackie virus expressed as virus titer (black squares with solid line). In each graph is also depicted time course of virus titer after the exposure to uncoated glass coverslips (black circles with dashed line). Error bars indicate standard deviations of three independent experiments

To assess the virucidal potential of hybrid coatings against other enveloped, nonenveloped, RNA and DNA viruses we selected dengue virus, coxsackievirus, influenza virus and herpes simplex virus. Droplet of 10 μl of each virus was applied on glass coverslips, incubated up to 4 h, titer of recovered virus was determined as specified in Methods section depending on the virus, and plotted in the same graph as titer of recovered virus from untreated glass coverslips (Fig. 6). Very quick initial virucidal effect of hybrid coatings was achieved against dengue virus with 1.1 log_10_TCID_50_ reduction after 30 min exposure, but followed with only additional 1.1 log_10_TCID_50_ decline in 4 h. Different course of exposure experiment to hybrid coatings was observed in the case of herpes simplex virus. Similarly 1.0 log_10_TCID_50_ decrease of HSV-1 titer was achieved in first 30 min, but complete inactivation was accomplished after 4 h. Only small decrease (0.7 log_10_TCID_50_) of influenza virus titer when compared with untreated control was achieved after 4 h of exposure to hybrid coating. Even smaller virucidal effect of hybrid coatings than against influenza virus was observed against coxsackie B3 virus, where titers of recovered virus from coated coverslips were on average only 0.2 log_10_TCID_50_ lower than titers of recovered virus from uncoated coverslips.

## Discussion

Development of broad-spectrum antimicrobial surface coating would represent significant help in the battle against hospital-acquired infections. We recently reported new type of hybrid coating containing silver and copper with strong antibacterial effect against variety of commonly occurring bacteria in hospital including *Staphylococcus aureus* and its methicillin-resistant variant [6]. To find out the potential antiviral effect, we decided to expose several different viruses to coated glass coverslips and poly(methyl methacrylate) wells in 96-well culture plates.

Our general observation is that the properties of prepared hybrid coatings depend more on the sol synthesis and the subsequent curing method and less on the types of substrate. This fact was further confirmed by the examination of chemical and mechanical resistance on different types of substrate [6, 49]. The thickness of coatings depends on the method of curing as coatings cured at 150 °C appear to be thinner than those cured at 90 °C and, than those cured by photopolymerization. Photopolymerized coatings showed worse mechanical properties than heatpolymerized coatings. These observations need to be further elucidated. According to SEM images, the surface of the glasses was homogeneously covered with the hybrid coatings. The coatings were visible only in the areas where they were mechanically disturbed. A very smooth surface without any visible defects was confirmed also by AFM measurement [49]. We assume that several round-shaped pores were created by gases that evolved at the substrate-coating boundary. Pores are clearly visible in Fig. 2 and 3. Nevertheless, the adhesion of the coatings on glass substrates was very good in both cases of tested polymerizations. There were not identified any places with disrupted adhesion or places containing uncollapsed bubbles with any of the methods used.

Thorough toxicological analysis of potential adverse effects is essential for successful implementation of hybrid coating in health care settings. In general, Ag^I+^, Cu^II+^ and Zn^II+^ ions are more toxic than their corresponding nanoparticles Ag, CuO, ZnO [50]. We observed only negligible cytotoxicity effect on Vero and HeLa cells when exposed to washings from coated glass coverslips. Moreover, we did not observe any significant increase in cytotoxicity when washing the coated glass coverslips immediately or after four 4 h of incubation. This fact shows that the metal ions are immobilized in hybrid coating most likely due to ionic bonding and the leaching of metal ions does not increase significantly during the 4 h span. It corroborates previous release study from coated cotton swab when the biggest decrease of Ag^I+^ and Cu^II+^ amount occurred only after first washing cycle and after fifty washing cycles there still remained 40 % of Ag^I+^ and 20 % of Cu^II+^ ions in the coating [6]. Certain degree of metal ions release from hybrid coating is necessary otherwise it would impede the virucidal effect. Here, we have shown that this release is not toxic on two mammalian cell lines but further animal studies to address sensitization and/or irritation are needed.

The antiviral activities of hybrid coating were initially tested against HIV-1 on glass coverslips prepared by the same sol-gel method, which differed only in polymerization process that was either heat- or photoinitiated. Coatings prepared by either curing method showed strong anti-HIV-1 activity after 5 min and further intensified after 20 min. The bigger decrease in residual HIV-1 activity after 20 min than after 5 min of exposure of HIV-1 to hybrid coating containing Ag^I+^, Cu^II+^ and Zn^II+^ ions supports hypothesis that these ions have direct inhibition effect on HIV-1 virions. It is generally accepted that silver and copper ions can exert their biocidal power by directly lysing membranes or by binding to thiol groups of proteins [34, 51, 52]. Zinc was shown to have strong antifungal [53, 54] and antibacterial [55–58] effect. In addition, we have plenty evidence of zinc virucidal activity against rhinoviruses [59–61], respiratory syncytial virus [62], vaccinia virus [63], HSV [64] and HIV-1 [65] but exact virus inactivation mechanism is mostly unknown. Kim and coworkers [66] showed that zinc ions can inhibit protein tyrosine phosphatase albeit to much smaller extent that copper ions but by different mechanisms not related to active-site cytosine. Additional proposed mechanisms for HIV-1 inactivation by zinc include inhibition of HIV-1 protease [67] and inhibition of HIV-1 DNA to RNA transcription [65]. Strong evidence that silver nanoparticles inhibit HIV-1 infection by blocking viral entry, particularly gp120-CD4 interaction provided the work of Lara and coworkers [25]. They showed in cell-based fusion assay using Env expressing cells and CD4 expressing cells mixture that silver nanoparticles blocked cell fusion in dose-dependent manner. The CD4 binding domain of gp120 has three disulfide bonds that would represent good target to interact with the virion not only for silver ions but also for copper ions. From reaction with glutathione in an excess of copper in the living cells it is assumed that one Cu^II+^ ion can inactivate 3 thiol groups [34].

Next we coated PMMA 96-well plates and confirmed quick and increasing anti-HIV-1 effect during prolonged incubation, albeit we achieved on average only 99 % reduction in HIV-1 titer after 2 h of exposure to hybrid coatings. Better results with glass coverslip experiments can be explained by better adhesion of coatings to glass surfaces as documented by SEM analysis and primarily by the fact that HIV-1 droplet on glass coverslip was covered with another coverslip and thus was more tightly distributed on larger coated surface.

Finally, to determine the hybrid coating ability to inactivate other viruses, we tested four additional viruses representing double-stranded DNA virus, negative and positive sense single-stranded RNA virus and non-enveloped virus. Interestingly, we observed different time course of virus titer reduction for these viruses. Positive single-stranded RNA enveloped dengue virus type 2 had initially quick titer reduction in first 30 min, but it was not followed by complete inactivation even after 4 h of exposure. There are limited data in the literature about anti-dengue activity of coatings with or without metal ions and nanoparticles. Dengue virus type 2 infection was not suppressed by nanoscale silica platelets hybridized with silver nanoparticles but introduction of anionic sodium dodecyl sulfate efficiently reduced titer almost about three log_10_TCID_50_ [68]. Recently, Murugan et al. reported 80 % reduction of dengue virus type 2 replication by silver nanoparticles at concentration of 50 μg/ml [69]. Binding of dengue virions to host cell receptor is mediated through viral envelope glycoprotein E, which contains 12 strictly conserved cysteine residues forming six disulfide bonds, followed by receptor-mediated endocytosis. Several mammalian cell receptors have been shown to interact with dengue virions such as sulfated glycosaminoglycans, lectins that recognize carbohydrates, glycosphingolipid, proteins with chaperone activity and others [70], but the exact mechanism is still not fully elucidated. Recently, the importance of lipid composition of host cells and virion membrane was highlighted [71]. Quick dengue titer reduction in the first 30 min of our experiment could signal direct effect on viral membrane and/or disruption of disulfide bonds by metal ions in our hybrid coatings.

In case of double-stranded DNA HSV-1 we have seen different course of virus titer reduction than with dengue virus type 2. Initial one log_10_TCID_50_ reduction in titer in the first half hour was followed by complete inactivation after 4 h. HSV entry is complex event involving five glycoproteins and three alternative cellular receptors with heparan sulfate being the most important but not essential [72]. Baram-Pinto and coworkers prepared silver nanoparticles capped by mercaptoethane sulfonate that mimic polysulfonated heparan and showed that these modified nanopraticles block the attachment of the virus to host cells [26]. Moreover, unmodified silver nanoparticles after one hour preincubation with HSV-2 inhibited subsequent infection of Vero cells with EC_50_ = 25 μg/mL [27]. Based on our results, that HSV-1 is completely inactivated after extended contact with the coating, we can hypothesize additional mechanism of HSV-1 inactivation besides direct interaction with virion particle. One of the possible mechanisms of action could be disruption of viral double-stranded DNA (dsDNA) by metal ions. It has been shown that silver, zinc and especially copper ions have specific affinity for dsDNA [32, 34, 73]. This mechanism was postulated for inhibition of partially double stranded hepatitis B virus by silver nanoparticles, which were able to reduce extracellular hepatitis B virus DNA by more than 50 % and thus inhibit the formation of intracellular HBV RNA.

The effect of hybrid coating against influenza A virus lead to only 0.7 log_10_TCID_50_ reduction after 4 h of exposure. Inhibition of influenza A was previously reported with silver nanoparticles, but the strongest inhibitory effect was achieved at rather high concentration of 50 μg/ml of silver nanoparticles [30]. Copper oxide-based filter reduced influenza A virus titer by 1.77 ± 0.87 log_10_TCID_50_ during 40 min filtration [37]. Mechanism for silver or copper ions anti-influenza activity is unknown. Recently, functionalized gold nanoparticles with sialic acid-terminated glycerol dendron were found effective against influenza virus that was mediated by blocking the sialic acid receptor on host cell surface, and thus blocking the fusion step of influenza infection [74].

Hybrid coating exerted only negligible effect on non-enveloped positive single-stranded RNA coxsackie B3 virus. Similarly, previously reported rhinovirus type 2, member of the same *Picornaviridae* family as coxsackie viruses, exhibited only nonsignificant decrease after passing through copper oxide-based filter [37]. These results would support the explanation that our hybrid coatings with silver, copper and zinc ions act mainly directly on the virion surface of the enveloped viruses.

## Conclusions

In summary, we have characterized virucidal effect of hybrid coatings containing silver, copper and zinc cations against different viruses representing double-stranded DNA virus, negative and positive sense single-stranded RNA virus and non-enveloped virus. Our experiments showed good virucidal effect against enveloped viruses especially HIV and together with previously reported excellent antibacterial effect this hybrid coating has potential to provide antimicrobial protection on surfaces and materials in healthcare settings.

### Availability of supporting data

The data sets supporting the results of this article are included within the article.

### Ethics approval and consent to participate

Not applicable.

### Consent for publications

Not applicable.

## Abbreviations

AFM: atomic force microscopy
ATCC: American type culture collection
BPO: dibenzoylperoxide
DMEM: dulbecco’s modified eagle’s medium
FBS: fetal bovine serum
FTIR: fourier transform infrared spectroscopy
HAIs: healthcare–associated infections
HIV-1: human immunodeficiency virus type 1
HSV-1: herpes simplex virus type 1
IPTI: titanium isopropoxide
MDCK: madin-darby canine kidney
MMA: methylmethacrylate
ORMOSILs: organically-modified silicates
PBS: phosphate buffered saline
PMMA: poly(methyl methacrylate)
PMS: phenazine methosulphate
SEM: scanning electron microscope
TCID50: 50 % tissue culture infective dose
TEOS: tetraethylorthosilicate
TMOS: tetramethylorthosilicate
TMSPM: 3-(trimethoxysilyl)propyl methacrylate
TPCK: tosyl phenylalanyl chloromethyl ketone
XTT: 2,3-bis-(2-methoxy-4-nitro-5-sulfophenyl)-2H-tetrazolium-5-carboxanilide
